# Supplementary material for: Drivers and determinants of extreme humanitarian needs among Rohingya refugee households: Evidence from UNHCR’s multi-sectoral needs analysis
Source: PLoS One. 2025 Dec 1;20(12):e0331727. doi: 10.1371/journal.pone.0331727 (PMC12668494; doi:10.1371/journal.pone.0331727)
Supplement: S1 Table — (DOCX) [file pone.0331727.s001.docx]

**Supplementary Table 1A.** Prevalence of Extreme Education Needs among School Aged Children Disaggregated by Reason and Gender.

|  | **Prevalence (% [95%CI])** | | |
| --- | --- | --- | --- |
|  | **Boys (n=3,634)** | **Girls (n=3,570)** | **School Age Children (N=7,204)** |
| **Children in Extreme Educational Need** | **6.7% [5.8%-7.7%]** | **15.1% [13.7%-16.6%]** | **10.9% [10.0%-11.8%]** |
| Protection Risks While at or Traveling to School | 1.7% [1.3%-2.3%] | 4.8% [4.0%-5.7%] | 3.2% [2.7%-3.8%] |
| Working Instead or Required at Home to Support Household Income | 4.7% [4.0%-5.6%] | 7.2% [6.2%-8.4%] | 6.0% [5.3%-6.7%] |
| Underage Marriage or Early Pregnancy | 0.3% [0.1%-0.6%] | 3.1% [2.5%-3.8%] | 1.7% [1.3%-2.1%] |

**Supplementary Table 1B.** Odds of Extreme Education Needs among School Aged Children by Gender.

|  | **n (%)** | **Prevalence (% [95%CI])** | **OR [95%CI]; p-value** |
| --- | --- | --- | --- |
| **School Age Children** | 7,204 | 10.9 [10.0–11.8%] | – |
| Boys | 3,634 (50.4%) | 6.7% [5.8%–7.7%] | 1 (ref) |
| Girls | 3,570 (49.6%) | 15.1% [13.7%–16.6%] | 2.47 [95%CI: 2.06–2.96]; p<0.001 |
