# Supplementary material for: Drivers and determinants of extreme humanitarian needs among Rohingya refugee households: Evidence from UNHCR’s multi-sectoral needs analysis
Source: PLoS One. 2025 Dec 1;20(12):e0331727. doi: 10.1371/journal.pone.0331727 (PMC12668494; doi:10.1371/journal.pone.0331727)
Supplement: S2 Table — (DOCX) [file pone.0331727.s002.docx]

**Supplementary Table 2.** Distribution of households in extreme humanitarian need by number and combination of sectoral needs.

| **Sector(s)** | **n (%)** |
| --- | --- |
| **One Sector** | **943 (27.7%)** |
| Protection | 2 (0.1%) |
| Education | 381 (11.2%) |
| Health | 150 (4.4%) |
| WASH | 28 (0.8%) |
| Food Security | 265 (7.8%) |
| Shelter & NFIs | 117 (3.4%) |
| **Two Sectors** | **217 (6.4%)** |
| Protection/Health | 2 (0.1%) |
| Protection/Food Security | 1 (<0.0%) |
| Protection/Shelter & NFIs | 1 (<0.0%) |
| Education/Health | 42 (1.2%) |
| Education/WASH | 11 (0.3%) |
| Education/Food Security | 88 (2.6%) |
| Education/Shelter & NFIs | 19 (0.6%) |
| Health/WASH | 1 (<0.0%) |
| Health/Food Security | 15 (0.5%) |
| Health/Shelter & NFIs | 12 (0.3%) |
| WASH/Food Security | 6 (0.2%) |
| WASH/Shelter & NFIs | 2 (0.1%) |
| Food Security/Shelter & NFIs | 17 (0.5%) |
| **Three Sectors** | **44 (1.3%)** |
| Protection/Education/Health | 1 (<0.0%) |
| Protection/Education/Food Security | 2 (0.1%) |
| Protection/Education/Shelter & NFIs | 1 (<0.0%) |
| Education/Health/WASH | 1 (<0.0%) |
| Education/Health/Food Security | 12 (0.4%) |
| Education/Health/Shelter & NFIs | 4 (0.1%) |
| Education/WASH/Food Security | 2 (0.1%) |
| Education/WASH/Shelter & NFIs | 1 (<0.0%) |
| Education/Food Security/Shelter & NFIs | 18 (0.5%) |
| Health/WASH/Food Security | 1 (<0.0%) |
| Health/Food Security/Shelter & NFIs | 1 (<0.0%) |
| **Four Sectors** | **2 (0.1%)** |
| Education/Health/Food Security/Shelter & NFIs | 1 (<0.0%) |
| Health/WASH/Food Security/Shelter & NFIs | 1 (<0.0%) |
