# Supplementary material for: Drivers and determinants of extreme humanitarian needs among Rohingya refugee households: Evidence from UNHCR’s multi-sectoral needs analysis
Source: PLoS One. 2025 Dec 1;20(12):e0331727. doi: 10.1371/journal.pone.0331727 (PMC12668494; doi:10.1371/journal.pone.0331727)
Supplement: S3 Table — (DOCX) [file pone.0331727.s003.docx]

**Supplementary Table 3.** FEWS NET Food Security Matrix.

|  | **rCSI: 0-3** | | | **rCSI: 4-18** | | | **rCSI: 19+** | | |
| --- | --- | --- | --- | --- | --- | --- | --- | --- | --- |
| **FCS:** | Acceptable | Borderline | Poor | Acceptable | Borderline | Poor | Acceptable | Borderline | Poor |
| **HHS:** |  |  |  |  |  |  |  |  |  |
| 0 | 527 (15.5%) | 236 (6.9%) | 23 (0.7%) | 912 (26.8%) | 552 (16.2%) | 47 (1.4%) | 98 (2.9%) | 64 (1.9%) | 12 (0.4%) |
| 1 | 69 (2.0%) | 39 (1.2%) | 1 (0.0%) | 248 (7.3%) | 206 (6.1%) | 20 (0.6%) | 38 (1.1%) | 25 (0.7%) | 5 (0.1%) |
| 2-3 | 5 (0.1%) | 1 (0.0%) | 1 (0.0%) | 72 (2.1%) | 78 (2.3%) | 18 (0.5%) | 40 (1.2%) | 44 (1.3%) | 13 (0.4%) |
| 3 | 0 (0%) | 0 (0%) | 0 (0%) | 0 (0%) | 1 (0.0%) | 0 (0%) | 1 (0.0%) | 3 (0.1%) | 0 (0%) |
| 5-6 | 0 (0%) | 0 (0%) | 0 (0%) | 1 (0.0%) | 0 (0%) | 0 (0%) | 0 (0%) | 0 (0%) | 0 (0%) |
