# Supplementary material for: Drivers and determinants of extreme humanitarian needs among Rohingya refugee households: Evidence from UNHCR’s multi-sectoral needs analysis
Source: PLoS One. 2025 Dec 1;20(12):e0331727. doi: 10.1371/journal.pone.0331727 (PMC12668494; doi:10.1371/journal.pone.0331727)
Supplement: S4 Table — (DOCX) [file pone.0331727.s004.docx]

**Supplementary Table 4.** Sensitivity analysis: Household characteristics associated with the odds of extreme humanitarian needs after removal of overlapping criteria.

|  | **Unadjusted** | | **Adjusted** | | **Criteria Removed** |
| --- | --- | --- | --- | --- | --- |
|  | **OR (95%CI)** | **p-value** | **aOR (95%CI)** | **p-value** |  |
| **Head of Household Gender** |  |  |  |  | ***Health Sector: (ii)*** unmet health need for antenatal/postnatal support, safe delivery, or gender-based violence |
| Male | 1.00 | – | 1.00 | – |  |
| Female | **1.29 (1.08–1.54)** | **0.005*** | **1.68 (1.32–2.15)** | **<0.001*** |  |
| **Household Size** | **1.16 (1.12-1.20)** | **<0.001*** | **1.16 (1.12-1.21)** | **<0.001*** | **Shelter & Non-Food Items*: (ii)*** Household is severely overcrowding with more than three people per room. |
| **At least 1 Member in Psychosocial Distress** |  |  |  |  | ***Health Sector: (ii)*** unmet health need for mental health and psychosocial support (MHPSS). |
| No | 1.00 | – | 1.00 | – |  |
| Yes | **1.32 (1.14-1.54)** | **<0.0.01*** | **1.24 (1.06-1.45)** | **0.008*** |  |
