# Supplementary material for: Drivers and determinants of extreme humanitarian needs among Rohingya refugee households: Evidence from UNHCR’s multi-sectoral needs analysis
Source: PLoS One. 2025 Dec 1;20(12):e0331727. doi: 10.1371/journal.pone.0331727 (PMC12668494; doi:10.1371/journal.pone.0331727)
Supplement: S1 Fig — (DOCX) [file pone.0331727.s005.docx]

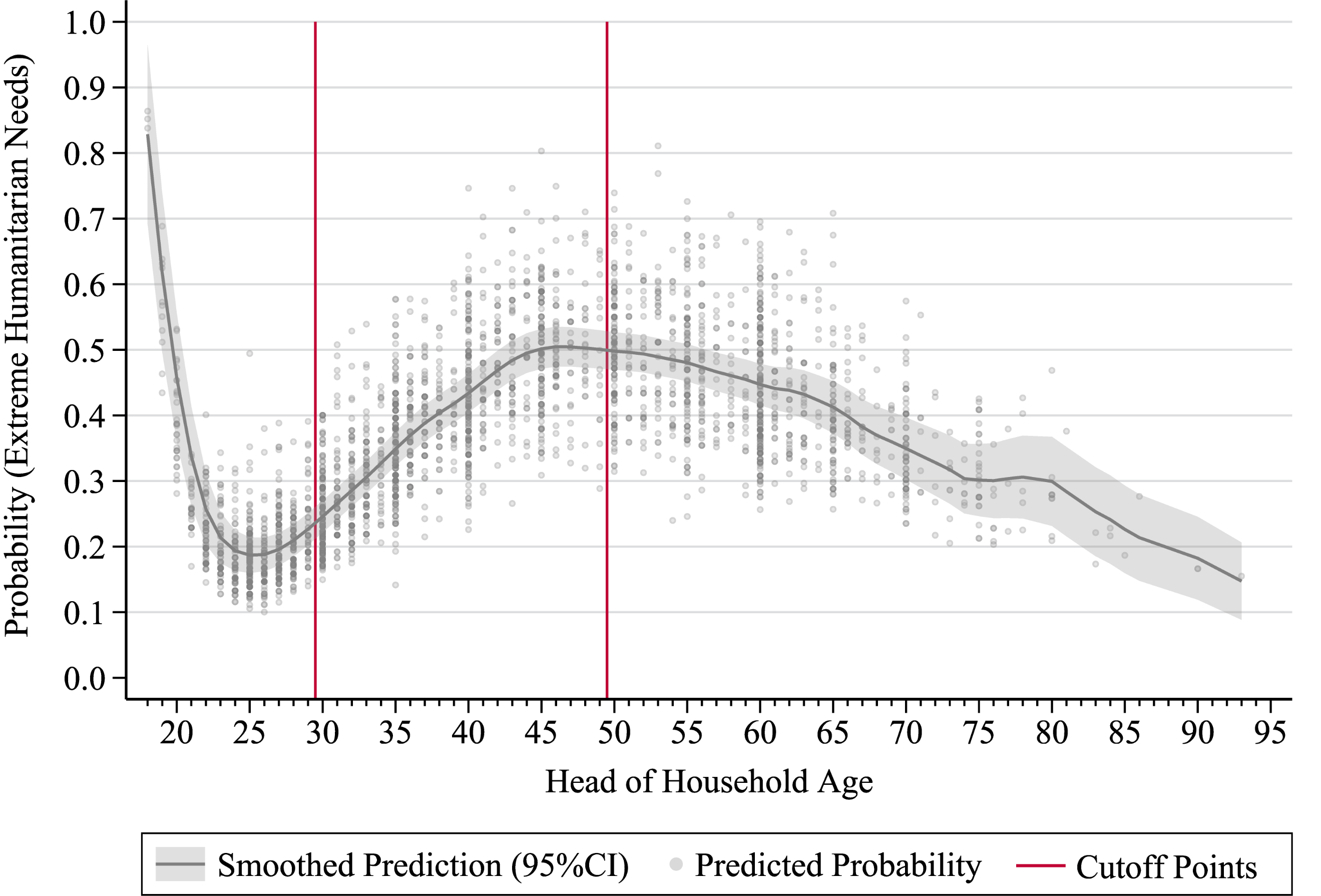
**Supplementary Fig 1A**. Adjusted predicted probability of extreme humanitarian needs by head of household age.

Plotted values represent probability estimates from adjusted logistic regression with head of household age entered as the best-fitting three-degree fractional polynomial. The polynomial terms and degrees were validated against a 5-year head of household age-group model

**
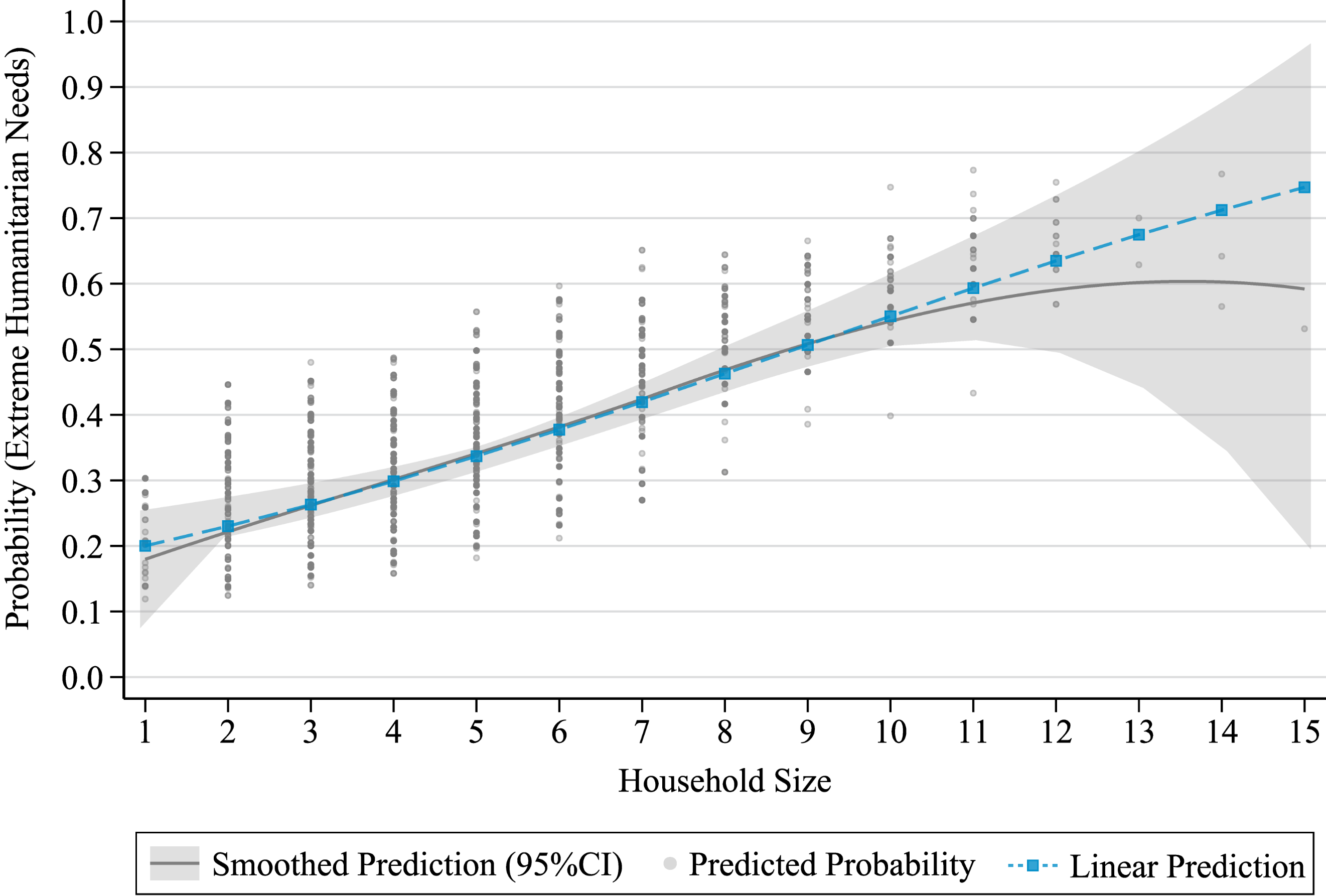
Supplementary Fig 1B**. Adjusted predicted probability of extreme humanitarian needs by household size.

Plotted values represent probability estimates from adjusted logistic regression with household size entered as the best-fitting two-degree fractional polynomial. The polynomial terms and degrees were validated against a categorical household size model.
