# Supplementary material for: Drivers and determinants of extreme humanitarian needs among Rohingya refugee households: Evidence from UNHCR’s multi-sectoral needs analysis
Source: PLoS One. 2025 Dec 1;20(12):e0331727. doi: 10.1371/journal.pone.0331727 (PMC12668494; doi:10.1371/journal.pone.0331727)
Supplement: Attachment — Submitted filename: Response to Reviewers.docx [file pone.0331727.s007.docx]

**Dear Editor and Reviewers,**

Thank you for your generous contributions, I sincerely appreciate the time you have spent to review my research manuscript.

I have revised the manuscript and submitted these files as per the recommendations.

Please see my responses to the review feedback below:

# Editor

1. **Please ensure that your manuscript meets PLOS ONE's style requirements, including those for file naming. The PLOS ONE style templates can be found at https://journals.plos.org/plosone/s/file?id=wjVg/PLOSOne_formatting_sample_main_body.pdf and https://journals.plos.org/plosone/s/file?id=ba62/PLOSOne_formatting_sample_title_authors_affiliations.pdf**

See markup for changes made and files renamed.

2. **Your ethics statement should only appear in the Methods section of your manuscript. If your ethics statement is written in any section besides the Methods, please delete it from any other section.**

The ethics statement is within the methods section. A slight alteration was made to move the data access date into the study design and setting section of the method.

3. **For studies involving third-party data, we encourage authors to share any data specific to their analyses that they can legally distribute. PLOS recognizes, however, that authors may be using third-party data they do not have the rights to share. When third-party data cannot be publicly shared, authors must provide all information necessary for interested researchers to apply to gain access to the data. (https://journals.plos.org/plosone/s/data-availability#loc-acceptable-data-access-restrictions)**

**For any third-party data that the authors cannot legally distribute, they should include the following information in their Data Availability Statement upon submission:**

1) **A description of the data set and the third-party source**
2) **If applicable, verification of permission to use the data set**
3) **Confirmation of whether the authors received any special privileges in accessing the data that other researchers would not have**
4) **All necessary contact information others would need to apply to gain access to the data**

The data availability statement has been amended to include the specific URL. This data source is also explicitly referenced in the methods section (see reference 10 in manuscript).

4. **We note you have included a table to which you do not refer in the text of your manuscript. Please ensure that you refer to Table 3 in your text; if accepted, production will need this reference to link the reader to the Table.**

Table 3 has been referred to in-text after the estimates provided for the first variable in that section. See manuscript markup.

5. **If the reviewer comments include a recommendation to cite specific previously published works, please review and evaluate these publications to determine whether they are relevant and should be cited. There is no requirement to cite these works unless the editor has indicated otherwise.**

Not applicable

6. **Please review your reference list to ensure that it is complete and correct. If you have cited papers that have been retracted, please include the rationale for doing so in the manuscript text, or remove these references and replace them with relevant current references. Any changes to the reference list should be mentioned in the rebuttal letter that accompanies your revised manuscript. If you need to cite a retracted article, indicate the article’s retracted status in the References list and also include a citation and full reference for the retraction notice.**

All citations have been validated and formatted as per requirements

# Reviewer 1

1. **On page 4, lines 75-76, pls clarify the 95% confidence level used, but the margin of error was set to 10%? Should it not be 5%, at 95% confidence level?**

This is based on the sampling strategy designed by the external parties that conduced the primary data collection. The confidence interval (z-score of 1.96) and margin of error (plus or minus 10%) are different components used to calculated the sample size rather than the reciprocal of each other.

The primary objective of the original data collection was to inform camp-specific gaps for a wide range of outcomes to monitor the response. The use of 10% margin of error in the sample size calculation was used to ensure that 95% of the time the camp-specific result had a minimum level of precision (+/- 10%) irrespective of the prevalence within the specific camp.

The margin of error is also different to the alpha threshold (5%) used to conclude statistical significance during hypothesis testing.

2. **On page 7, lines 176-177 - i suggest reiterating how extreme humanitarian need is defined. While this was mentioned in the methods section, it helps to mention again in the results to guide readers.**

I respectfully disagree and do not believe that restating how the outcome was defined is required in both the methods and results section. This aspect is more relevant to the variables portion of the methods section where it has been made available if the reader needs to scroll back and re-read. I think this change would also disrupt the logical flow of the manuscript.

3. **Given that education is the top driver of humanitarian need, i wonder why households with school-going age population not included as a predictor? The paper did not offer any recommendation, moving forward. How do we address the education need of the young in humanitarian setting? I encourage the authors to reflect on this.**

This variable would likely induce the measurement bias from criterion overlap between this predictor (school age children) and the education related indicators (At least one school aged child not attending/not enrolled or learning in an unsafe environment for extreme reasons) used to assign the outcome (see Reviewer 2: Response 3 for more details).

This exact variable was considered (households with school-age children) but rejected due to homogeneity as it categorised almost all households. A similar linear variable (number of school age children within the household) was considered, as were other variables relating to dependents (school age children, those with a disability or the elderly). These were all too highly correlated with increasing household size, which was selected in preference to mitigate criterion overlap and due to superiority in a mutually adjusted model. In essence, household size was actually a better predictor than the number of school age children or another similar variable related to the household composition.

Regarding recommendations for the education sector, this is an excellent point and exactly where the research is headed. The results prompt a much more granular sector-specific analysis of the education sector. What may seem like a surface level acknowledgment is actually a lead-in to a prospective paper that is underway. This work will inform specific educational interventions that require more careful elucidation by age-group and gender. These interventions are not deliverable without adequate funding. Additionally, in the context of this paper regarding broader humanitarian needs, I have addressed the underlying dynamics of the educational sector in relation to its role as both an independent driver and symptom of the living conditions that exacerbate negative household coping strategies. From this perspective, redressing the educational needs is inextricably linked to addressing the household’s humanitarian needs and improving the living conditions within the camps.

# Reviewer 2

1. **“Data” is a plural term. Please check subject – verb agreement throughout the manuscript.**

See markup for changes where required.

2. **Add study design to title or abstract**

No changes were made as the study design was stated in the first sentence of the abstract’s methods section:

“Data was sourced from the 2023 Joint Multi-Sectoral Needs Assessment (J-MSNA) - a representative **cross-sectional survey** of Rohingya refugee households”

3. **Describe potential sources of bias and any efforts to address them**

I believe that these efforts are inherently addressed by the sampling design that was described and statistical analysis that was conducted without the need for a revision in the manuscript to accommodate an exhaustively list of bias. For clarity, specific sources of bias were addressed as follows:

Stratified simple random sampling and survey weights were used to control for selection bias (ie. random selection of households within each camp and survey weights to address the differential probabilities of household selection between the camps).

The age-gender structure of the sample was contrasted against the population to evidence any potential sampling bias. No such bias was evidence that otherwise require acknowledgment or further statistical remediation.

Multivariable logistic regression to control for confounding. Particularly the age and gender structure demonstrated minor confounding after adjustment which is specifically highlighted in the results section.

Potential effect heterogeneity (effect modification) was specifically investigated between marital status and head of household gender. This was based on the odds associated with female headed households that may be due to concentrated effects among single women. As per the results section, stratified analysis was used to confirm effect homogeneity in this particular circumstance.

The criterion overlap is another structural issue due to household characteristics being used as both indicators (such as severe overcrowded) and predictors (such as increasing household size) of the outcome (households in extreme humanitarian need). This issue was addressed through two techniques. Firstly, distancing the predictive variables from the indicator variables by using more general household characteristics rather than those explicitly used as logic to assign the outcome. Secondly, sensitivity analysis was conducted using all indicators except those with the potential to overlap. This confirmed the presence of an independent association and provided a counterfactual to contrast the effect sizes reported in the main analysis against to ensure no problematic distortion arose from criterion overlap.

4. **What was the refusal rate for participating in the survey? Consider a flow diagram to show how many approached, how many eligible, how many responded, how man included in the analysis?**

The response rate was >98% (3400 households sampled, 3465 households invited).

Given the scope of results presented, I do not believe an additional figure is required or would add additional depth to the method. A research terms of reference document and methodological overview that covers these queries are publicly available and referenced in-text (see references 16 and 17 of the manuscript).

Instead of a flow diagram, an individual level age-gender diagram of sampled participants versus the population registered in UNHCRs database was provided (Figure 1). I believe has more utility through informing the generalisability of the results than extending the methods section which is described in adequate details with further details available elsewhere for those inclined. I am happy to address any specific queries from readers through correspondence.

5. **Were there missing data? How was missing data addressed?**

No data was missing and therefore no acknowledgement of missing data was provided.

6. **Construct the conclusion to reflect what can be concluded from this study. Other commentary belongs in the Discussion section.**

I agree and have toned down the statements made in the conclusion. The updated text recites the main findings, applies their implications within the research context of the challenges facing the humanitarian response and provides a call to actions for the international community to ensure that the response is adequately funded as per the humanitarian needs.

**Regards,**

**Harry J Wilson (B.BMed.Sci, MPH, MIDI, MGH)**
